# A cross-sectional survey of knowledge, attitude and practice (KAP) towards COVID-19 pandemic among the Syrian residents

**DOI:** 10.1186/s12889-021-10353-3

**Published:** 2021-02-05

**Authors:** Sanaa Al ahdab

**Affiliations:** 1 Department of Pharmacology and Toxicology, Faculty of Pharmacy, Al-Rasheed International University for Science and Technology, Ghabagheb, Daraa’, Syrian Arab Republic; 2 Department of Pharmacology and Toxicology, Faculty of Pharmacy, Al-Baath University, Homs, Syrian Arab Republic

**Keywords:** COVID-19, Knowledge, Attitude and practice, Syria, Pandemic

## Abstract

**Background:**

Effective COVID-19 pandemic management requires adequate understanding of factors that influence behavioral changes. This study aims to assess knowledge, attitudes and practices towards COVID-19 among Syrians in a post-conflict context.

**Method:**

A cross sectional web-based survey was conducted on the Syrian residents of 16 years and above. It contains questions on knowledge, attitudes and practices (KAP) with respect to COVID-19. Participants’ demographic characteristics are recorded and analyzed. The study is conducted during the global outbreak of COVID-19.

**Results:**

A total of 706 participants (female, 444; male, 262) were enrolled. This study included 405 participants aged between 16 and 29, 204 aged between 30 and 49, and 97 aged above 60 years. There were 642 who have a university degree and 61 who have high school degree. Among the participants 253 were students, 316 were employed, 75 work as freelancers, and 62 were unemployed. Results showed that overall knowledge score towards the disease was about 60% (mean score 3.54 ± 1.20; range 0–6). Knowledge scores significantly differed across age groups (*P* < 0.05), education levels (*P* = 0.001), and occupations (*P* < 0.05). Attitude and practice scores were 2.45 ± 0.81 (range 0–4), 5.90 ± 1.52 (range 0–8), respectively. Attitude scores were significantly different between males and females (*P* < 0.05), whereas practice scores varied significantly across gender (*P* < 0.05), age groups (*P* = 0.01), education levels (*P* = 0.015), occupations (*P* < 0.05), and according to knowledge score (*P* = 0.000). Results from multiple linear regression indicated that lower knowledge scores were significantly associated with lower education level (*P* < 0.05), whereas poor preventive practices were common among male, young and unemployed participants with significance levels of *P* < 0.01, *P* = .000, *P* < 0.01, respectively.

**Conclusion:**

The findings of this study suggest that the Syrian residents demonstrate modest knowledge, attitudes and practices towards COVID-19 at the time of its global outbreak. Efforts should be directed towards raising the awareness of the disease to improve their practices in the current COVID-19 pandemic, as well as for future epidemics.

**Supplementary Information:**

The online version contains supplementary material available at 10.1186/s12889-021-10353-3.

## Background

Since the discovery of the novel coronavirus disease 2019 (abbreviated as COVID-19) in China in December 2019, the disease has spread quickly across the globe. Despite its strong foothold in the region, particularly in Lebanon, Jordan, and Iran, the spread of COVID-19 in Syria was late. The first COVID-19 laboratory-confirmed case was reported on 22 March [1], with the first fatality reported a week later [2]. The disease has spread quickly, and by July 03, 2020, there were 328 laboratory-confirmed cases and 10 deaths as reported by the Syrian Ministry of Health. There were some concerns on the accurateness of these figures, partially due to limited testing capacity of COVID-19 owing to limited resources and sanctions imposed on Syria [3]. Syrian health authorities used purchased and gift kits to conduct approximately one hundred tests daily in Damascus [4] that reached around 2.700 tests as of May 8 [5]. Moreover, many cases were probably not reported because tests were available only to people who showed clear symptoms or had contact with confirmed cases or deaths. COVID-19 is expected to be of a great challenge to the Syrian war-torn health system because of its fragile health system aggravated by the lack of sufficient equipment [6] and the loss of around 70% health workers [7].

In 2018, the Syrian government spent less than 1% of its total expenditure on health [8]. In 2019, the number of beds was disproportionally distributed between cities and among private and government hospitals with an average number of population of 699 per bed [8]. The total number of intensive care unit (ICU) beds was approximately 650 in hospitals all over Syria (excluding the city of Idlib) [8]. Although private hospitals provide relatively better healthcare services compared to public hospitals, they suffered from similar shortages and problems as government hospitals did.

To stem the spread of the disease, the Syrian government has imposed similar measures to those adopted by other countries. On March 24, a curfew was declared from 6 pm to 6 am, in addition to the closure of shops, markets, parks, and public transport. Classes at schools and universities were curtailed, travel between cities was banned, and all incoming and outgoing flights were also suspended. In addition, lockdown was imposed on areas where confirmed or suspected infections or deaths were reported. On May 26, the government decided to gradually loosen curfew restrictions in order to bring people back to work and salvage the economy.

Prior evidence on good knowledge, attitudes and practices (KAP) among the public are essential for successful control and outbreak prevention of pandemics; such as severe acute respiratory syndrome (SARS) in 2003 [9–13], H5N1 epidemic [14], Swine Flu [15, 16], H1N1 [17, 18], and COVID-19 [19–21]. To the best of our knowledge, there is no published study among the Syrian residents about KAP towards pandemics. This paper aims at investigating the KAP of the Syrian residents towards COVID-19 pandemic in a post-conflict context. While the Syrian health authorities aspire to curtail the spread of the pandemic with their control measures, their effectiveness will depend on the individual and societal awareness and practices. It is interesting to find out how similar measures can be perceived differently in different contexts; particularly in the Syrian special case where the underdeveloped health system associates the conflict.

## Materials and methods

### Design and settings

To measure the KAP towards COVID-19 of the Syrian residents, a web-based questionnaire was developed for this study and used for data collection from 5 April to 9 April, during the second week after the lockdown of the Syrian territories. The questionnaire was posted on social media platforms (Facebook and WhatsApp). Privacy of the original post was set as “public” to enable more people to participate in the questionnaire.

### Participants

Persons who were aged 16 years or more, agreed to participate in the study. Although the target participants were local Syrian citizens, Syrians who reside outside Syria were also eligible for this survey. However, the latter’s answers were excluded because this study focused only on people who reside in Syria.

### Questionnaire

The questionnaire was based on a similar questionnaire on COVID-19 [19] and other questionnaires on H1N1 [16, 22], and Severe Acute Respiratory Syndrome, SARS [11] as well as concepts theories of health behaviors [23]. The questionnaire takes about 5 min to complete. The questionnaire language is Arabic. This questionnaire [see Additional file 1] collected basic demographic data on age, gender, education level, employment status, and residents above 60 or younger than 16 in the house. Questions on knowledge aimed to assess general knowledge on clinical presentations of COVID-19 and the severity of the disease (Q1-Q6). These questions were answered as on a yes/no basis with an additional “maybe” option. Questions on attitudes (A1-A4) were about the agreement with the measures adopted to prevent the spread of the pandemic COVID-19 and the confidence in winning the battle against COVID-19. Questions on practices were used to assess the individuals’ compliance and behaviors during the quarantine (P1-P8). A summary of questions assessed is shown in Table 2.

### Statistical analysis

Independent sample *t-*test, one-way analysis of variance (ANOVA) test of significance, and multiple linear regression were used to examine the relation between the demographic characteristics and variables. A Pearson correlation analysis was used to compare correlations between variables. All statistical analysis was performed using IBM SPSS 24 for Windows.

## Results

A total number of 825 participants completed the survey questionnaire. After excluding 119 respondents who live outside Syria, the final sample consisted of 706 participants [see Additional file 2]. Among the final sample, 444 (62.9%) were female, 405 (57.4%) aged between 16 and 29 years, 642 (90.9%) were either at higher education level or above, 316 (44.8%) were employed in private or public occupations and 516 (73.1%) had residents in their houses aged above 60 and/ or younger than 6 years. Demographic characteristics are shown in Table 1.

**Table 1:** Demographic characteristics of participants and the score of COVID-19 knowledge by demographic variables

Characteristics		Number of participants (%)	Knowledge score (mean ± standard deviation)	***P*** value
Gender	Male	262 (37.1)	3.46 ± 1.25	> 0.05
	Female	444 (62.9)	3.59 ± 1.17	
Age-group (years)	16–29	405 (57.4)	3.43 ± 1.16	< 0.05
	30–49	204 (28.9)	3.71 ± 1.29	
	50+	97 (13.7)	3.62 ± 1.15	
Education	Elementary School	3 (0.4)	2.00 ± 1.73	0.001
	High school	61 (8.6)	3.08 ± 1.26	
	Degree	642 (90.9)	3.59 ± 1.18	
Occupation	Unemployed	62 (8.8)	3.54 ± 1.36	< 0.05
	student	253 (35.8)	3.39 ± 1.10	
	Free lancer	75 (10.6)	3.76 ± 1.25	
	Employed	316 (44.8)	3.62 ± 1.23	
Residents above 60 or younger than 16 in the house	No	190 (26.9)	3.52 ± 1.12	> 0.05
	Yes	516 (73.1)	3.55 ± 1.23	
**Knowledge of COVID-19 score**		**706 (100)**	**3.54 ± 1.20**	

This study shows that the Syrians’ Basic knowledge of COVID-19 is moderate. The correct answer rates of the 6 questions on the COVID-19 knowledge questions ranged between 22.7–85% (Table 2). The mean knowledge score was 3.54 (SD: 1.20, range: 0–6) suggesting an overall 59% (3.54/6*100) correct rate on this knowledge test. Knowledge scores significantly differed across age groups (*P* < 0.05), education levels (*P* = 0.001), and occupations (*P* < 0.05) (Table 1). Knowledge regarding COVID-19 symptoms was the highest (85.1%), whereas perception of the severity of COVID-19 was the lowest (22.7%). Having a child under 16 or aged persons above 60 did not make a statistical difference in the COVID-19 knowledge. Furthermore, no statistical difference was found between genders with regard to knowledge scores (Table 1).

**Table 2:** Summary of Questions for Knowledge, Attitudes and Practices towards Pandemic COVID-19. Percentages represent the correct answers

Questions	
**Knowledge**	
Q1.The main clinical symptoms of COVID-19 are fever, fatigue, dry cough, and myalgia (85.1%)	
Q2. Symptoms of COVID-19 are similar to the common symptoms of flu (55.2%)	
Q3. COVID-19 infection causes severe symptoms in all patients (22.7%)	
Q4. Persons with COVID-2019 can infect the virus to others when a fever is not present (56.2%)	
Q5. COVID-19 infection causes serious disease (44.6%)	
Q6. Although there is no proven cure for Corona disease, the available treatments lead to recovery (35.3%)	
**Attitudes**	
A1. Do you think school closure is an effective way of preventing the spread of the disease? (91.5%)	
A2. Do you think curfew is an effective way of preventing the spread of the disease? (78.2%)	
A3. Do you think that COVID-19 will spread widely in Syria? (15.4%)	
A4. Do you think that COVID-19 will be successfully controlled? (60.1%)	
**Practices during quarantine**	
**Avoidance behavior**	
P1. Avoid crowded places (92.5%)	
P2. Avoid travel by taxi (80%)	
P3. Avoid shaking hands (82%)	
**Personal Habits Practice**	
P4. Practice better hygiene than before (90.8%)	
P5. Use disinfectants (ethanol) (73.5%)	
P6. Wear facemasks (27.9%)	
P7. Wash hands more often (95.8%)	
P8. Have a balanced diet (47.9%)	

For the attitudes, the majority of respondents believed that school closure and curfew were effective in controlling the spread of COVID-19; scores were 91.5 and 78.2%, respectively (Table 2). Only 15.4% of the respondents expected the pandemic to spread in Syria, whereas about 60% had confidence that COVID-19 will eventually be controlled. Attitude scores were significantly different between males and females (*P* < 0.05) (Table 3).

**Table 3:** Attitudes towards COVID-19 by demographic variables

Characteristics		Attitude score (mean ± standard deviation)	***P*** value
Gender	Male	2.54 ± 0.87	< 0.05
	Female	2.40 ± 0.77	
Age-group (years)	16–29	2.49 ± 0.77	> 0.05
	30–49	2.40 ± 0.85	
	50+	2.40 ± 0.90	
Education	Elementary School	2.33 ± 0.57	> 0.05
	High school	2.38 ± 0.93	
	Degree	2.46 ± 0.80	
Occupation	Unemployed	2.29 ± 0.87	> 0.05
	Student	2.48 ± 0.76	
	Free lancer	2.55 ± 0.82	
	Employed	2.44 ± 0.84	
Residents above 60 or younger than 16 in the house	No	2.49 ± 0.76	> 0.05
	Yes	2.44 ± 0.84	
**Attitude score**		**2.45 ± 0.81**	

In addition, the mean practice score was 5.90 (SD: 1.52, range from 0 to 8). The highest practice score was 75% (6/8*100) by participants aged over 50 years. Practice scores varied significantly across gender (*P* < 0.05), age groups (*P* = 0.01), education levels (*P* = 0.015), occupations (*P* < 0.05), and according to knowledge score (*P* = 0.000) (Table 4). More than 90% of participants avoided crowded places and practiced better hygiene after than before the quarantine, whereas only 27.9% of participants wore facemasks during the quarantine (Table 2).

**Table 4:** The score of practices towards COVID-19 by demographic variables

Characteristics		Practice score (mean ± standard deviation)	***P*** value
Gender	Male	5.75 ± 1.64	< 0.05
	Female	6 ± 1.44	
Age-group (years)	16–29	5.76 ± 1.55	0.01
	30–49	6.04 ± 1.49	
	50+	6.22 ± 1.42	
Education	Elementary School	5.33 ± 2.30	0.015
	High school	5.38 ± 1.82	
	Degree	5.96 ± 1.48	
Occupation	Unemployed	5.47 ± 1.77	< 0.05
	Student	5.81 ± 1.55	
	Free lancer	5.99 ± 1.61	
	Employed	6.04 ± 1.42	
Residents above 60 or younger than 16 in the house	No	5.92 ± 1.42	> 0.05
	Yes	5.90 ± 1.56	
**Practice score**		**5.90 ± 1.52**	

The results from multiple linear regression analysis of variables that score poor on KAP indicators show that the education level of elementary school (β: -1.70, *P* < 0.05) was significantly associated with lower knowledge score, whereas females had lower attitude score (vs. males, β: -0.14, *P* < 0.05). Furthermore, male gender (vs. female, β: -0.36, *P* < 0.01) aged between 16 and 29 years (β: -0.42, *P* = 0.000), and unemployed (β: -0.66, *P* < 0.01) were predictors of poor practice score (Table 5).

**Table 5:** Results of multiple linear regression on factors associated with poor COVID-19 knowledge, attitude and practice

Variable	Coefficient (β)	Standard error	t	***P*** value	95.0% Confidence Interval for B	
					Lower Bound	Upper Bound
**Knowledge**						
Age (16–29)	− 0.19-	0.11	−1.65-	> 0.05	− 0.41-	0.04
Education (Elementary School)	−1.70-	0.70	−2.44-	< 0.05	−3.06-	−0.33-
Occupation (Students)	−0.13-	0.12	−1.06-	> 0.05	− 0.36-	0.11
**Attitude**						
Gender (female)	−0.14-	0.06	−2.25-	< 0.05	−0.27-	− 0.02-
**Practices**						
Gender (male)	−0.36-	0.12	−2.97-	< 0.01	−0.59-	−0.12-
Age (16–29)	−0.42-	0.12	−3.59-	.000	−0.65-	−0.19-
Education (elementary school)	−0.45-	0.88	−0.51-	> 0.05	−2.18-	1.29
Occupation (unemployed)	−0.66-	0.21	−3.17-	< 0.01	−1.06-	−0.25-

There was positive and significant correlation between knowledge-practice, knowledge-attitudes, and attitude-practice. The correlation coefficients were (0.198, 0.204, and 0.210, respectively. *P* < 0.01) as shown in (Table 6).

**Table 6:** correlations between scores of knowledge, attitudes and practices towards COVID-19

Variable(s)	Knowledge	Attitudes	Practices
Knowledge	1	–	–
Attitudes	0.204^a^	1	–
Practices	0.198^a^	0.210^a^	1

^a^Correlation is significant at the 0.01 level (2-tailed)

## Discussion

This study is expected to be the first to examine the KAP towards COVID-19 among the Syrian residents. In this predominantly female and well-educated population, the overall correct rate of around 60% on the knowledge questions indicated that most respondents have modest knowledge about COVID-19.

The majority of the participants believed that the school closure and curfew were effective in preventing the spread of COVID-19. However, 15.4% of participants believed that the virus would spread in Syria and only 60% had confidence that COVID-19 will eventually be successfully controlled. Despite this, the practices of the Syrian residents were cautious: 92.5% avoided crowded places and shaking hands and 90.8% practiced better hygiene than before the quarantine, like washing hands and using disinfectants. Surprisingly, however, only 27.9% wore facemasks when leaving homes. This study also analyzed the characteristics of KAP towards COVID-19 and identified some demographic factors associated with KAP; these findings are useful for public health policy-makers and health workers to identify and target people for COVID-19 prevention and health education in case of future outbreaks.

The finding of a modest knowledge score of COVID-19 of participants, although the majority were well-educated, was unexpected. Their scores were lower than their counterparts in China [19] and in Iran [20] which showed an overall correct rate of 90% among the Chinese and Iranian populations, respectively. However, Syrians scored higher in terms of their COVID-19 knowledge compared to the population of northern Thailand in the early period of the outbreak [21]. In Thailand, 73.4% had poor knowledge towards COVID-19 [21]. The finding of moderate knowledge score was probably because Syrian residents have not experienced previous pandemics such as H1N1 or SARS. In addition, this survey was conducted during the very early stages of COVID-19 in Syria when the country was not seriously affected by the outbreak, with the number of laboratory confirmed COVID-19 cases of only 19 (Ministry of Health), the lowest among other countries in the region. This underlines the importance of the Syrian health authorities providing consistent clear updates and information about the emerging virus as well as the need to continuously assess whether their messages are being understood within the community.

Since the World Health Organization declaration of the COVID-19 outbreak to be pandemic on March 12, 2020 [24], countries around the world have implemented different measures to prevent further spread of the virus. Many countries have applied school closure as a response to COVID-19 according to UNESCO [25]. Other restrictions also included curfews and stay-at-home orders. Similarly, the Syrian government has also closed schools and enforced curfew while allowing some essential businesses to open. The vast majority of Syrians included in this survey believed that these measures were effective against COVID-19.

Furthermore, compared to KAP studies in China and Iran, Syrians under study were less optimistic about the disease control than the Chinese [19] and Iranians [20]. The underlying reason could be related to the quality of the Syrian health care system that has seriously been affected by the nine-year war, and further deteriorated due to sanctions [26]. Moreover, efforts to aid the COVID-19 control were limited due to shortages of medical workers and medical materials [4].

Although attitudes towards COVID-19 were unassertive, Syrians took precautions to prevent infection by COVID-19: not going to crowded places and practiced better hygiene with an overall practice score of about 74%. This may be attributed to their doubts on the ability of the health care system to accommodate them if they are infected. However, this score was lower than other practice scores towards COVID-19 documented among Chinese [19] and Iranians [20]. Unfortunately, the present study showed that only approximately 30% wore facemasks when going outside home. This could be primarily attributed to the unavailability of quality facemasks and the surge in their prices which increased by 6 to 10 folds particularly during the time of this survey.

The potentially poor documented practices were associated with younger age males. This is in agreement with the findings of similar COVID-19 studies in China [19] and Iran [20]. The finding that women were more likely to practice non-pharmaceutical health behavior (e.g., hand washing) is also consistent with a previous study on SARS pandemic [27]. Furthermore, this study is in agreement with previous evidence that late adolescents were also more likely to engage in risk taking behaviors [28]. It is unsurprising that unemployment was also associated with poor practices because of having less exposure to the COVID-19 virus. Therefore, the unemployed are expected to be less adherent to health safety measures of keeping social distancing and wearing facemasks.

This study provides evidence on the positive and significant correlations between knowledge-attitudes, knowledge-practice, and attitudes- practices among the respondents (Pearson correlation coefficient approximately 0.2). This reaffirms that better knowledge and attitudes associate with better practices. Similar levels of association between these variables were documented in a previous study on H1N1 pandemic [29]. Therefore, health authorities should not only intensify their efforts to improve health services but also give equal importance to raising people awareness and knowledge towards COVID-19.

## Conclusion

In summary, this study provides insights into Syrians’ knowledge, attitudes and practices towards COVID-19 during the quarantine in a post-conflict context. This is expected to help Syrian health authorities formulate suitable measures to counter the spread of COVID-19 and develop best practices for future epidemics. Yet, the successfulness and impact of current measures on controlling COVID-19 are still unclear and debatable.

This study has some limitations. First, the use of an internet-based survey may overlook people who do not have internet access or Facebook/WhatsApp accounts. Second, the sample may be over-representative by respondents with higher education levels compared to others with lower education levels. This may inflate the overall results, as these groups are probably more knowledgeable and have better practices towards pandemics in general. Third, this study does not distinguish between different income groups while investigating KAP. Nevertheless, participants’ income could have important impact on their attitudes and practices towards the pandemic. Fourth, this study does not examine the impact of knowledge on both attitude and practices. All these limitations represent venues for further new research.

Future research could also focus on how different sources of information affect respondents’ knowledge. That is, future research can try to find the best source that delivers reliable information to avoid panic or false sense of security among the general public. In addition, it may be worth investigating how infected people react to re-infection news and if they would change their attitudes and practices in response to this news.

The results of this study may not be generalizable to other countries or cities that have experienced previous severe epidemics such as SARS. This study presents a unique reference for pandemic cautious behavioral response to COVID-19 in a post-conflict context. Nevertheless, it was challenging to finish data collection within a short period (5 days) before lockdown measures were eased off.

## Supplementary Information

**Additional file 1.** Questionnaire-English version. This table represents an English version of the questionnaire

**Additional file 2.** Resopnse.variables.scores. Description of data: Data related to demographic characteristics, responses to KAP questions, and KAP scores. Responses are represented as 0 or 1. KAP scores represent the sum up of responses to each group questions.

## Data Availability

The datasets used and analysed during the current study are available from the corresponding author on reasonable request.

## Abbreviation

COVID-19: Coronavirus disease 2019
