# Supplementary material for: A cross-sectional survey of knowledge, attitude and practice (KAP) towards COVID-19 pandemic among the Syrian residents
Source: BMC Public Health. 2021 Feb 5;21:296. doi: 10.1186/s12889-021-10353-3 (PMC7863039; doi:10.1186/s12889-021-10353-3)
Supplement: Supplementary file 1 — Additional file 1. Questionnaire-English version. This table represents an English version of the questionnaire [file 12889_2021_10353_MOESM1_ESM.docx]

**Questionnaire used for Knowledge, Attitudes and Practices towards Pandemic COVID-19.**

| Questions |
| --- |
| **Knowledge (yes, no, maybe)** |
| Q1.The main clinical symptoms of COVID-19 are fever, fatigue, dry cough, and myalgia |
| Q2. Symptoms of COVID-19 are similar to the common symptoms of flu |
| Q3. COVID-19 infection causes severe symptoms in all patients |
| Q4. Persons with COVID-2019 can infect the virus to others when a fever is not present |
| Q5. COVID-19 infection causes a serious disease |
| Q6. Although there is no proven cure for Corona disease, the available treatments lead to recovery |
| **Attitudes (yes, no, maybe)** |
| A1. Do you think school closure is an effective way for preventing the spread of the disease? |
| A2. Do you think curfew is effective way of preventing the spread of the disease? |
| A3. Do you think that COVID-19 will spread widely in Syria? |
| A4. Do you think that COVID-19 will be successfully controlled? |
| **Practices during quarantine (yes, no, sometimes)** |
| **Avoidance behavior** |
| P1. Avoid crowded places |
| P2. Avoid travel by taxi |
| P3. Avoid shaking hands |
| **Personal Habits Practice** |
| P4. Practice better hygiene than before |
| P5. Use disinfectants |
| P6. Wear facemask |
| P7. Wash hands more often |
| P8. Have a balanced diet |
